# Economic Evaluation of the Metabolic and Bariatric Surgery Initiative for Patients With Obesity and Comorbid Diabetes in Queensland

**DOI:** 10.1002/osp4.70144

**Published:** 2026-04-20

**Authors:** Paul Scuffham, Katie Wykes, Jody Paxton

**Affiliations:** ^1^ Centre for Applied Health Economics School of Medicine & Dentistry Griffith University Gold Coast Australia; ^2^ Healthcare Improvement Unit Clinical Excellence Queensland Queensland Health Herston, Queensland Australia

**Keywords:** Australia, cost‐effectiveness, diabetes, patient prioritization, Roux‐en‐Y, sleeve gastrectomy, weight loss

## Abstract

**Background:**

Metabolic and bariatric surgery is the most effective long‐term treatment for patients with obesity; however, access to publicly funded surgery in Australia has been limited despite strong evidence of clinical benefit. The Bariatric Surgery Initiative (BSI) was established to improve equitable access for people with obesity and type 2 diabetes, but its long‐term economic value has not been evaluated. This study assessed the cost‐effectiveness of metabolic and bariatric surgery compared with usual medical care to inform health‐system decision‐making.

**Methods:**

A Markov model was developed to simulate health outcomes and costs over the rest of life for patients with BSI. The model had five health states representing BMI classes. Costs obtained from the BSI for the surgical procedure were used and long‐term costs for BMI classes were drawn from the literature. Quality of life (utility) weights were obtained from the BSI as were outcomes at 12 months following surgery. A Usual Care comparison group was developed from the literature and long‐term costs were applied to both groups depending on the BMI class. Incremental cost‐effectiveness ratios were estimated and sensitivity analyses were undertaken from the perspective of the Australian public health system.

**Results:**

The base‐case analysis demonstrated that MBS was the preferred strategy that is, better health outcomes (2.5 QALYs gained) and lower costs (cost‐savings of $67,000). Scenario analyses identified that younger age groups have greater health benefits and higher BMI classes have greater cost‐savings.

**Conclusions:**

MBS is a cost‐effective intervention for patients with obesity class 2 and diabetes. The BSI was an efficient service that provided the necessary information to develop the local evidence.

## 1 Introduction

Metabolic and bariatric surgery (MBS) is the most effective long‐term treatment for obesity [1]. MBS results in sustained weight loss, reduction of obesity‐related complications, improved quality of life and improved survival [2, 3, 4]. However, metabolic and bariatric surgery in Australia has been largely performed in the private health care sector and has remained inaccessible to many eligible patients due to socioeconomic and geographic inequality [5, 6].

The Queensland metabolic and bariatric surgery service, known as the Bariatric Surgery Initiative (BSI), was created by Queensland Health in 2019 to provide equitable access to surgery for people with obesity and type 2 diabetes. The BSI was established as a state‐wide service allowing patients from anywhere in Queensland to be referred for MBS in public hospitals regardless of location or ethnicity. The BSI involved inclusion/exclusion criteria, a central referral hub and a patient prioritization scoring system to identify people most likely to have the greatest benefit. The BSI was highly effective in reducing body weight and improving health‐related quality of life in adults with obesity and type‐2 diabetes [7].

This surgery program would have significant resource and cost implications for Queensland's healthcare system, but its long‐term value for money had not been evaluated. There are large up‐front costs for surgery and the benefits, including cost‐offsets, do not occur for some time in the future. However, failure to provide the surgery will lead to substantial future healthcare costs for the complications of obesity and diabetes. From a policy perspective, decisions to allocate resources are generally based on health economic analyses, with cost‐effectiveness analysis being the most common approach [8]. Consequently, the aim was to carry out a cost‐utility analysis (CUA) comparing MBS with usual medical care among people with obesity and comorbid diabetes.

CUA, a type of cost‐effectiveness analysis, is a preferred and best method for assessing value for money and thereby enables decisions about allocating scarce health resources. CUA compares the cost of different interventions with their health outcomes measured in quality adjusted life years (QALY). QALYs are calculated by estimating the total life years gained from a procedure and weighting each year of life to reflect the quality of life in that year [9]. The results of a CUA are summarized as the ratio of the difference in costs between two interventions to the difference in their effectiveness (QALYs) and expressed as the incremental cost‐effectiveness ratio (ICER). If the ICER is less than society's willingness to pay for an additional unit of health benefit (QALY in this case), then that intervention is deemed cost‐effective with acceptable value for money. In Australia, the upper limit ICER threshold has been reported to be approximately AU$64,000 [10].

The aim of this study was to evaluate the long‐term cost‐effectiveness of MBS in Queensland. MBS undertaken with either Roux‐en‐Y gastric bypass or sleeve gastrectomy was compared with usual care (UC; i.e., medical management) to estimate the longer‐term costs and health outcomes. This current economic evaluation is based on a real‐world data drawn from the BSI where possible and supplemented with data from the literature and administrative data.

## 2 Methods

Participants eligible for inclusion in the BSI were people with type 2 diabetes with an HbA1c > 7% despite treatment with Metformin (or alternative) plus at least one other diabetes medication, a body mass index (BMI) ≥ 35 kg/m^2^ and aged between 18 and 65 years. Exclusion criteria included factors such as current alcohol or drug dependency, end‐stage renal failure, malignancies under active treatment, previous MBS and more; the full list was previously published in the supporting information [7]. The central referral hub established a process for referrals that required pathology and patient data, patient education materials including a video that the patient was required to watch, and an assessment by an expert multidisciplinary medical team. Referrals were accepted from any Queensland hospital and health service outpatient specialists, with additional referral pathways for Aboriginal and Torres Strait Islander patients through Aboriginal Health Workers, Aboriginal Community Controlled Health Organizations, Aboriginal and Torres Strait Islander Health Units, and rural or remote general practitioners where specialist services were unavailable. The central referral hub demonstrated the equity in access to MBS and the remaining gaps for targeting [11].

The patient prioritization tool was a pilot scoring system to identify the patients likely to have the greatest expected clinical benefit. Patient characteristics, such as weight, body mass index (BMI), glycated hemoglobin, diabetes medications, surgical risk and obesity‐related comorbidities, were formed into an index on a scale where 0 = minimal benefit and 100 = maximum benefit. The BSI received 292 referrals between 2017 and 2020 with follow‐up at 12 months. Higher scores were associated with younger age, higher BMI, and insulin use. Following surgery, higher‐scoring patients were more likely to discontinue oral diabetes medications, and glycated hemoglobin improvement was fourfold greater among those scoring 70–79 than lower scores of 20–29. The outcomes of patient prioritization are reported elsewhere [12].

**FIGURE 1:**
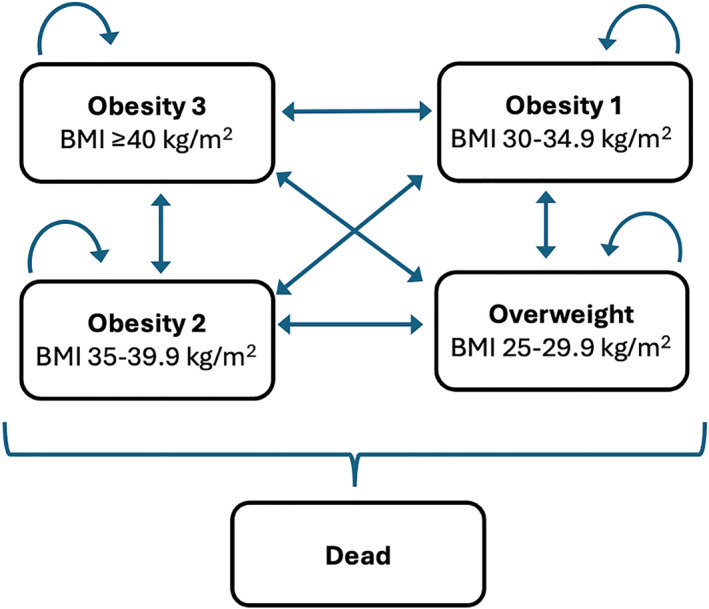
Markov model structure; note that patients could move into the death state from any of the other BMI based health states.

The CUA was conducted from a publicly funded health system perspective with a time horizon of 90 years. The MBS procedures of Roux‐en‐Y gastric bypass or sleeve gastrectomy were compared with UC (i.e., medical management) in terms of their cost‐effectiveness. UC in Queensland involved regular outpatient visits to an endocrinology and diabetes clinic for diabetes management and education. This is important for this group with poorly controlled diabetes. A multidisciplinary team was also involved to provide weight loss management. Multidisciplinary care included lifestyle interventions with dietary modification guided by dietitians, exercise advice guided by physiotherapists, and behavioral interventions provided by psychologists. For the CUA, a mathematical model (a Markov model) was constructed to estimate the incremental cost per quality‐adjusted life year (QALY) gained—the ICER. A Markov model was chosen as it was an ideal tool for reflecting the natural history of chronic diseases such as obesity [13]. Five health states were included based on the BMI category, plus the absorbing state of the dead (Figure 1). Costs and utility weights were assigned to each health state, as well as probabilities of transitioning from one health state to another.

The Markov model was built in the software TreeAge (TreeAge Pro, Healthcare Version 2024, TreeAge Software LLC). The structure of the model as shown in Figure 1 was similar to the model reported by Robbie et al. [14]; patients were categorized into mutually exclusive health states based on the body mass index (BMI) classes; these were 25–29.9 kg/m^2^ for overweight (OW), 30–34.9 kg/m^2^ for Class I Obesity (OB1), 35–39.9 kg/m^2^ for Class II Obesity (OB2) and 40+ for Class III Obesity (OB3) [15]. MBS is an intervention for obesity rather than diabetes per se; as such, the change in class of obesity was modeled, similar to other studies [16, 17] rather than complications of obesity such as diabetes or other various diseases [18, 19, 20, 21]. “Normal weight” was excluded from the model because of limited evidence on outcomes following MBS; excess skin following rapid weight loss can result in classification within the overweight category rather than the normal weight category, without affecting management of metabolic syndrome.

The health states, such as alive with OB2, were specified in the Markov model; transitioning between health states (e.g., moving from one BMI class to another, and back again) over time is a key feature of these models. Costs and quality of life (utility) weights were assigned to each health state and over the time horizon of the model the costs and QALYs accumulate. The direct healthcare costs included the cost of surgery itself, pre‐ and post‐surgery costs, and medication use, and the costs for UC were the direct healthcare costs associated with each health state. Indirect costs such as loss of productivity and out‐of‐pocket expenses were not included.

The patient cohort in the model had the demographics approximating the patients who went through the BSI program. The mean initial age of the cohort matched the BSI cohort of 52.4 ± 8.1 years. For the base‐case, the proportions of patients referred to the BSI at the time of referral were 0.5% in OB1, 20.0% in OB2 and 79.5% in OB3. The treatment effect of surgery on BMI reduction for the first 12 months was directly informed from the BSI program [7]. Long‐term outcomes were drawn from the STAMPEDE trial [22]; that trial reported outcomes over 5 years for patients with diabetes, most of whom were in Class 2 Obesity. The data for the surgical group from the STAMPEDE trial informed the probabilities for transitioning between health states beyond the initial 12 months post‐surgery.

The treatment effect of UC was derived from the “Look AHEAD” study comparing diabetes support and education with UC [23]. Outcomes for 8 years were reported in that study. The UC group outcomes from that study were used to inform the transition probabilities in the current study. Of note, the outcomes for the UC group at 5 years were similar to the outcomes reported for the UC group in the STAMPEDE trial [22, 23].

Simplifying assumptions around the probability of diabetes were made. In the UC group, all patients who had diabetes were assumed to continue to have diabetes (i.e., there was no remission of diabetes for this group). Type 2 diabetes with HbA1c > 7% despite treatment with oral medications and/or insulin were entry criteria to the BSI. For the surgical group, the proportion at 12 months post‐surgery who were in remission of diabetes was assumed to remain static (i.e., 10.1%) [7].

The health state utilities were obtained from the BSI using the AQoL‐4D at baseline and follow‐up, and supplemented with population norms for the overweight BMI class [24]. The health state utilities at baseline were grouped into BMI class using the BSI data. The follow‐up at 12 months for the BSI provided health state utility for BMI class “overweight.” This provided direct estimates of health state utilities for OW, OB1, OB2 and OB3 (Table 1) and the utility weights were applied in both the surgery and UC arms of this analysis. Adjustments were included for age (i.e., −0.003 ± 0.002 per year of age) to reflect decreasing health‐related quality of life with increasing age [31, 32], and additional disutility of 0.05 was applied to diabetes to account for the disutility of insulin injections, anxiety around perceived potential risk of complications such as peripheral neuropathy, renal disease, macular degeneration and the consequences of these risks being realized [32, 33].

**TABLE 1:** Input parameters.

Parameter	Value	Std dev	Distribution	Values for sensitivity analyses	Source
Universal model parameters					
Initial age (years)	52.4	8.1	Triangular (min = 28.1; max = 65)	28; 65	[7]
Discount rate (annual)	5%			3%, 7%	[25]
Time horizon modeled (years)	40			25, 55	^a^
Initial BMI class:					
OW; BMI = 25–29.9	0.0%				[7]
OB1; BMI 30–34.9	0.5%			0%, 100%	[7]
OB2; BMI 35–39.9	20.0%			0%, 100%	[7]
OB3; BMI 40+	79.5%			0%, 100%	[7]
Relative risk of mortality with diabetes	1.54			1.33, 1.78	[26]
Mortality (ages 30–90 years)	0.0028			0.00053 (30 yrs)–0.15277 (90 yrs)	[27]^b^
Surgery parameters:					
Surgical mortality	0.005	0.01	Beta		[28]
% with diabetes at 12 months post‐surgery	10.1%			5.0%, 15%	[7]^c^
Change in obesity class—year 1:					
OB1 to OW	100%			Fixed	[7]
OB1 remain OB1	0%			Fixed	[7]
OB2 to OW	64%			Fixed	[7]
OB2 to OB1	31%			Fixed	[7]
OB2 remain OB2	5%			Fixed	[7]
OB3 to OW	34%			Fixed	[7]
OB3 to OB1	23%			Fixed	[7]
OB3 to OB2	19%			Fixed	[7]
OB3 remain OB3	24%			Fixed	[7]
Subsequent years					
Annual increase in BMI class	1.8%	0.6%	Beta	0.9%, 2.7%	[22]
Usual care parameters:					
Change in obesity class					
Annual increase in BMI class	6.03%	0.47%	Beta	3.0%, 9.0%	[23]^d^
Annual decrease in BMI class	5.57%	0.47%	Beta	2.8%, 8.4%	[23]^d^
% with diabetes	100%				
Quality of life parameters (utility weights):					
OW	0.782	0.212	Beta	0.570, 0.994	[7]^e^
OB1	0.730	0.229	Beta	0.501, 0.959	[7]^e^
OB2	0.599	0.252	Beta	0.347, 0.851	[7]^e^
OB3	0.550	0.275	Beta	0.275, 0.825	[7]^e^
Disutility for diabetes	0.116	0.034	Beta	0.082, 0.150	^e^
Disutility for each year of age	0.003	0.002	Beta	0.001, 0.005	^e^
Cost parameters:					
Surgery 1st year					
OB1/OB2	$15,734	$12,886	Gamma	$11,014, $28,620	BSI
OB3	$18,242	$21,165	Gamma	$12,769, $39,407	BSI^f^
Medical management^g^					
OW	$6762	$15,568	Gamma	$4,733, $22,330	[29]
OB1	$7574	$16,008	Gamma	$5,302, $23,582	[29]
OB2/OB3	$8926	$18,599	Gamma	$6,248, $27,525	[29]
Universal cost parameters					
Diabetes costs	$3997	$248	Gamma	$2,798, $5196	[30]
Annual cost increase of diabetes	5%				^a^
Annual cost inflator for OB2	5%			0%, 10%	^a^
Annual cost inflator for OB3	10%			0%, 20%	^a^

Abbreviations: BMI, body mass index; BSI, data derived directly from the bariatric surgery initiative; OB1, class 1 obesity; OB2, class 2 obesity; OB3, class 3 obesity; OW, overweight.

^a^ Assumed parameter value.

^b^ Life tables—weighted average of males and females. Values reported in this table are for those aged 30 and 90 displayed here as examples.

^c^ For simplicity, the percentage using insulin was a proxy for diabetes and assumed to be constant for the rest of life.

^d^ Assumed that the annual change in weight was reflective of the change in BMI class.

^e^ Calculated from BSI patient data using the AQoL‐4D [24].

^f^ Same values used for surgical patients from year 2 onwards.

^g^ Costs for treatments or complications are expected to increase by 5% annually.

All‐cause mortality was taken from Australian life‐tables for each year of life [27]. An annual increased risk of death from complications of diabetes of 1.54 above the population risk was assigned to diabetes in both groups [26]. For the BSI group, there was an additional risk of surgical mortality assigned; a parameter of 0.5% was used to reflect this risk [28].

Costs for surgery were estimated from a sample of *n* = 86 who underwent MBS in the BSI where micro‐costing data from the Queensland Hospital Admitted Patient Data Collection were linked and provided by Queensland Health; the data included detailed micro‐costs for the six months before surgery and 12 months following surgery, including the costs for surgery. The costs for RYGB and SG were $14,889 (± $18,433, *n* = 59) and $14,591 (± $10,473, *n* = 27) respectively with 80% of participants receiving RYGB and 20% SG. A weighted average was used for the cost of surgery.

The costs for the second‐year post‐surgery, and the costs for UC were dependent on the health state and the presence of diabetes. An annual cost was assigned to each health state based on a costing study of 243,894 Australians stratified by BMI class [29], see Table 1. The total direct healthcare costs (hospital inpatient, emergency department, outpatient medical, and prescription drugs) across overweight, OB1, and OB2/3 categories were used, after adjustment for inflation, in the current study [29]. Costs were 12% higher in OB1 than in the overweight group and 18% higher in OB2/3 than in OB1 [29]. For this evaluation, annual cost increases of 5% for OB2 and 10% for OB3 were applied ‐ both lower than the 18% differential reported in Buchmueller et al. [29]. Additional costs for the management of diabetes and its complications were included based on an AusDiab study of 6101 participants and the values reported for cases of known diabetes [30]. The AusDiab study reported direct healthcare costs that included ambulatory services (e.g., visits to general practitioners, outpatient consultations, emergency department visits, hospital inpatient costs, medications, medical consumables such as glucose meters) and direct non‐healthcare costs (e.g., transport to hospital, home services such as home help/support) [30]. An initial value of $3997 annually was assigned for diabetes management (i.e., the AusDiab study direct healthcare costs converted to 2022/23 values) and assumed to increase by 5% per year of diabetes.

The model used an annual cycle with a discount rate of 5% for both costs and health outcomes [25]. All costs were converted to, reported in 2022/23 Australian dollars (AU$1 ≈ US0.71) using the CCEMG–EPPI Center Cost Converter v.1.7 [34]. A willingness to pay (WTP) threshold of A$64,000 was used as the value of a QALY [35]; this value was used to determine cost‐effectiveness and to estimate net monetary benefits (NMB).

The parameters used in the base‐case analysis are shown below in Table 1. Where possible and feasible, the distributions of data were incorporated into the model. This enabled probabilistic sensitivity analysis (PSA) to be undertaken to illustrate the likelihood that MBS is good value for money. One‐way sensitivity analyses were undertaken to identify the key factors affecting the results. For the one‐way sensitivity analysis, transition probabilities were varied by ± 50%, utility and disutility weights varied by one standard deviation, and due to the highly skewed distribution of costs, costs were varied from 70% of the base value to one standard deviation above the base value. Finally, a scenario analysis was undertaken to identify the cost‐effectiveness and NMB of restricting MBS to different age groups and class 2 versus class 3 obesity. The CHEERS 2022 checklist was used for the reporting the economic evaluation [36].

Ethics approvals were received from the Metro South Hospital and Health Service and Griffith University Human Research Ethics Committees (Project HREC/18/QPAH/427).

## 3 Results

MBS resulted in greater QALYs per patient due to more patients moving to lower BMI levels and improved survival relative to UC (Table 2). MBS resulted in lower direct healthcare costs per patient over the subsequent 40 years. The cost‐effectiveness ratio showed that surgery was a more effective and less costly strategy compared with UC as health outcomes were better, and costs were lower. The proportion of simulated patients in each health state over time, and the costs over time, are illustrated in the Markov traces (Supporting Information S1).

**TABLE 2:** Base case analysis for cost, QALY and cost‐effectiveness ratios.

Base case	UC	Surgery
Total costs	$138,821	$90,154
Total QALYs	5.942	7.022
Incremental cost		−$48,667
Incremental QALYs		1.080
ICER		Dominated

*Note:* Dominated: intervention improves health outcomes and reduces costs.

Abbreviations: ICER, incremental cost‐effectiveness ratio; QALY, quality‐adjusted life year; UC, usual care.

**FIGURE 2:**
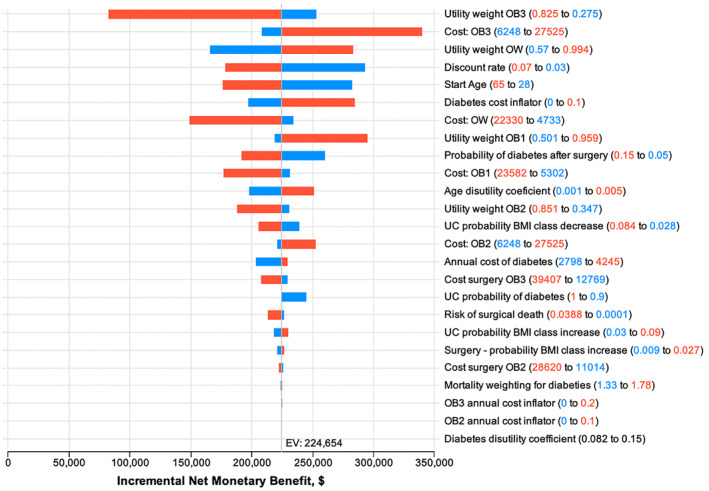
One‐way sensitivity analysis, using tornado diagram. Red numbers were the upper input value and the red bars indicated the effect on the net monetary benefit of using the upper input value; vice versa for the blue numbers and blue bars. OB1, class 1 obesity; OB2, class 2 obesity; OB3, class 3 obesity; OW, overweight state; UC, usual care.

Figure 2 illustrates the effect on the cost‐effectiveness ratio of varying each parameter in a one‐way sensitivity analysis and depicted as a tornado diagram. The utility weight and costs for the health state OB3 had the greatest impact on the results; however, both these factors encompassed substantial ranges. For example, the utility weight for OB3, obtained directly from the BSI using the AQoL‐4D [24], ranged from a low of 0.275 to a high of 0.825 on a scale of 0–1; costs for managing people in the OB3 class ranged from $6248 to $27,525 annually [29]. Using a high utility weight value for OB3 substantially reduces the NMB, whereas a low value allows for great improvements in the quality of life from weight loss. The cost of OW and the utility weight of OB1 had the opposite effects compared to the costs and utility weight of OB3 (e.g., higher cost of OW reduces the NMB). Other factors were largely unremarkable and varied around the base case values.

**FIGURE 3:**
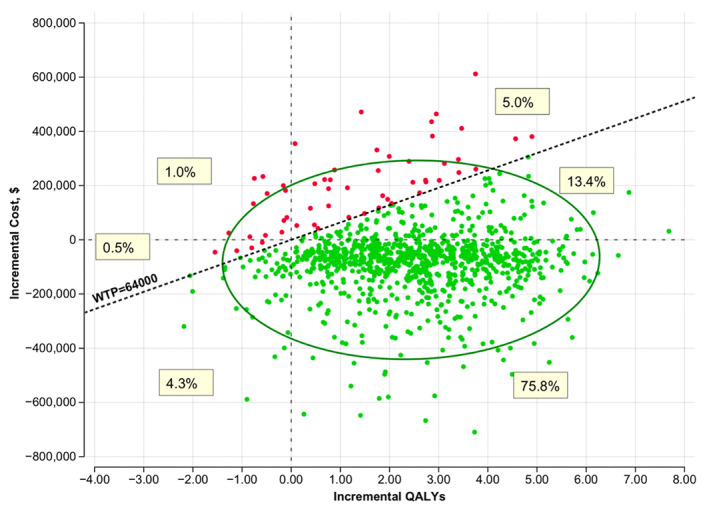
Cost‐effectiveness scatter plot for surgery versus usual care. This graph represents a scatter plot of simulations from the Markov model. The ellipse represents the 95% credible interval. Green dots indicate surgery as the optimal strategy.

In the probabilistic sensitivity analysis (Figure 3), 75.8% of the simulations fell into the cost‐saving and QALY gain quadrant (i.e., the lower right), 13.4% of the simulations had increased costs and gains in QALYs (the upper right) below the WTP threshold of $64,000. Another 4.3% of the simulations were lower cost and lower QALYs (lower left quadrant). Overall, 93.5% of the simulations were below the WTP threshold and 80.7% likelihood of being cost saving.

A series of scenario analyses were undertaken to identify if there is an age‐group and BMI class who might have greater benefits and lower costs (i.e., a greater return on investment). Various initial ages and BMI classes were analyzed (Table 3). In all scenarios, surgery resulted in lower costs and greater health outcomes compared with UC; that is, surgery is an economically superior choice. The greatest gains were in the younger age groups and the higher BMI (OB3). People aged 30 years with a BMI class of OB3 had the greatest gain in health benefits and the greatest reductions in costs over the rest of life (to age 90 years) compared with UC. Of note is that those in OB2 who underwent surgery compared with those in UC had greater gains in quality of life, whereas those in OB3 had greater cost‐savings.

**TABLE 3:** Scenario analysis of different initial ages and OB classes.

Initial age	Initial BMI class	Incremental costs	Incremental QALYs	ICER	NMB
30	OB2	−$69,406	2.206	Dominated	$225,183
30	OB3	−$74,286	1.986	Dominated	$169,237
40	OB2	−$45,531	2.537	Dominated	$193,227
40	OB3	−$49,059	2.238	Dominated	$140,823
50	OB2	−$28,857	2.761	Dominated	$152,023
50	OB3	−$31,014	2.382	Dominated	$106,095
60	OB2	−$17,431	2.763	Dominated	$105,076
60	OB3	−$18,248	2.322	Dominated	$ 68,644

Abbreviations: ICER, Incremental cost effectiveness ratio; NMB, incremental net monetary benefits.

## 4 Discussion

The results of the analysis implied that metabolic and bariatric surgical procedures were cost‐effective. An initial age of 52 years was used in the main analyses, but cost‐effectiveness was evident for all ages analyzed (from age 30–60). The evaluation modeled BMI classes up to age 90 years. The scenario analysis suggested that MBS should be offered to patients who are 30 years old and/or living with class 2 obesity or class 3 obesity. Maximum value was identified in the younger age‐groups with class 2 obesity (Table 3). Although the costs of surgery were greater than UC initially, the cost‐savings accrued starting the year after surgery. Improvements in quality of life commenced in the year of surgery. MBS was associated with substantial clinical benefits in terms of BMI reduction and improved quality‐adjusted survival, and cost‐savings compared with UC.

The cost‐savings would be much higher if all diabetes‐related complications attributable to overweight and obesity were taken into consideration. A previous report by the Australian Food and Nutrition Monitoring Unit estimated that a reduction in body weight of 1–5 kg could potentially reduce health system costs for Type 2 diabetes and complications by between $8.5 million and $43.7 million, respectively [37].

A previous modeled cost‐effectiveness analysis estimated much greater costs and cost‐savings than the current study [17]. That study did not have real‐world data available, and the analysis was based on data from international studies. The differences in costs were substantial—for example, the cost for surgery (including the index hospital stay) in that study ranged from $44,000 to $55,000, whereas the costs for surgery in the BSI averaged $17,740 (SD: $19,509) and were derived from the micro‐costs captured from the time of referral to 12 months post‐surgery. Similarly, the reduction in weight in the BSI was almost twice that used in the previous study (i.e., 28 kg in the BSI vs. 14.5 kg for Roux‐en‐Y and 16.2 kg for sleeve gastrectomy). Other inputs, including the utility weights, also differed substantially; in the James et al. 2017 study, population norms with a negative weighting of 0.003 for each kilogram of weight for those with a BMI > 30 kg/m^2^ were used, whereas the current study measured quality of life and derived utility weights for each BMI class. Overall, the high‐level conclusions remain the same—that is, MBS is a cost‐saving and quality of life improving procedure compared with UC.

A recent UK study reported the cost per QALY gained of £10,126 for Roux‐en‐Y gastric bypass compared with no weight management program [16]; however, that study did not target those with diabetes and therefore the cost‐savings and health benefits are likely to be underestimated. This finding is supported by two recent independent UK studies on MBS targeting individuals with diabetes and obesity, both of which report surgery as the preferred strategy (i.e., lower treatment costs, reduced diabetes‐related complications and better health outcomes) [18, 38]. Other studies confirmed that MBS was the preferred strategy compared with UC when the target population is those with obesity and diabetes [19, 20, 39] or obesity and a major comorbidity [40].

A strength of this study was that the BSI enabled data on patient characteristics, clinical outcomes, costs and quality of life to be captured; however, the BSI was a pilot implementation trial with no control group. Nevertheless, the BSI enabled quality of life scores, costs and changes in BMI class to be calculated for each BMI class for the 12 months following surgery. The limitations of this were that data for the period following the 12 months after surgery and for the UC group had to be drawn from the literature; international studies on the long‐term outcomes of surgery and UC for people with obesity and diabetes were used for estimating changes in BMI class for the rest of life [22, 23]. In the absence of long‐term Australian data, the results from international studies were assumed to directly transfer to Australia. Large Australian studies on costs of obesity and costs of diabetes were used for this purpose [29, 30]; however, those studies relied on data that was somewhat dated—for example, the costs of diabetes published by Lee et al. in 2013 used data from 2004 to 2005 [30], and the costs of obesity reported by Buchmueller et al. in 2015 used administrative data from 2006 to 2009 [29]. Currently, these are the best available data from large studies specific to Australia; however clinical practice and patterns in the use of health services is likely to have developed and changed considerably over the last 15–20 years and therefore those data may hold less relevancy. Nevertheless, the estimates of costs from those studies were applied to both the surgery and UC groups in the current study and as such, although the costs might not be accurate to reflect current practice, using any other numbers applied to both groups might result in small changes in the estimated cost‐savings it will have little impact on the conclusions.

Other limitations were that all potential obesity‐diabetes related complications were not included in the model and generalized assumptions were made around increasing costs and risks of death for those with class 2 or class 3 obesity and diabetes. However, those assumptions were tested in sensitivity analyses and had little impact on the results; as such those factors might have underestimated the cost‐savings and benefits from the surgery.

In conclusion, MBS is cost‐effective, substantially improved patient's quality of life and survival, and is expected to lead to significant cost‐savings for the health care system.

## Author Contributions

P.S. was responsible for designing the methodology, developing the Markov model and preparing the draft manuscript. J.P. and K.W. were responsible for the BSI program, data curation, review and editing of the manuscript.

## Funding

P.S. received funding from Queensland Health for the evaluation of this healthcare service. P.S. was the recipient of a NHMRC Senior Research Fellowship (SRF‐B, GNT1136923). J.P. and K.W. were administrators of the BSI employed by Queensland Health.

## Conflicts of Interest

The authors declare no conflicts of interest.

## Supporting information


Supporting Information S1

## Data Availability

The clinical data collected during the BSI used is government owned. An application for data access under the Public Health Act 2005 must be approved by the data custodian and the Director General of Queensland Health. To access the Statewide Bariatric Surgery Data Collection, email the request to the Executive Director, Healthcare Improvement Unit at HAAT@health.qld.gov.au. For further information: https://www.health.qld.gov.au/hsu/pha. Supporting Information S1 data used in the analysis are in the public domain from published literature.
